# Sharing the love: networking amongst cytometrists

**DOI:** 10.1186/1476-9255-12-S1-O2

**Published:** 2015-04-16

**Authors:** Derek Davies

**Affiliations:** 1 FACS Laboratory, London Research Institute, Cancer Research UK, 44 Lincolns Inn Fields, London, WC2A 3LY, UK

## 

Core Technology Laboratories, Shared Resource Laboratories, Platform Technologies, Shared Facilities – whatever name they are given, laboratories housing specific, generally expensive, equipment are very common in all areas of biomedical research. Focus with these laboratories is rightly given to the local end users who need to be trained in the specific technique to enable them to produce an experimental result. The journey from idea to end product requires inputs on many levels and this may not always be possible at a laboratory level. Flow cytometry is a relatively well-established and mature technology and the principles underlying cell analysis and sorting are well-understood. However, access to teaching of these principles is not always easy and there can be reliance on the equipment manufacturers to provide this. Establishment of local users groups for flow cytometrists in the UK in the mid 1980’s meant that like-minded researchers could meet and discuss ideas, problems and new opportunities. Cytometry in the UK was served for many years by the Royal Microscopical Society until the formation in 2006 of *flowcytometry***UK**. This organisation aims to act more as a focal point for users of the technology, allowing local groups to access a larger audience, bringing a national structure to allow people to connect more easily and facilitating knowledge and idea exchange amongst not only core facilities but those having very specific aims. One of the challenges for technological ‘societies’ is how to provide the most value without unnecessary duplication. We look forward to the participation of the Scottish Society for Cytomics and being able to work in partnership to help provide added value for members.

